# Small incision cataract surgery: tips for avoiding surgical complications

**Published:** 2008-03

**Authors:** Reeta Gurung, Albrecht Hennig

**Affiliations:** Deputy Medical Director, Tilganga Eye Centre, Kathmandu, Nepal. Email: reetagurung@gmail.com; Programme Director, Eastern Regional Eye Care Programme, Lahan, Nepal. Email: akhennig@gmx.net

**Figure:**
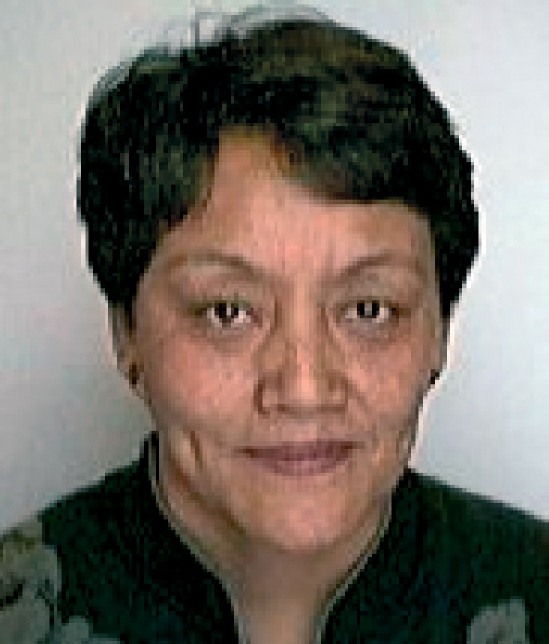


**Figure:**
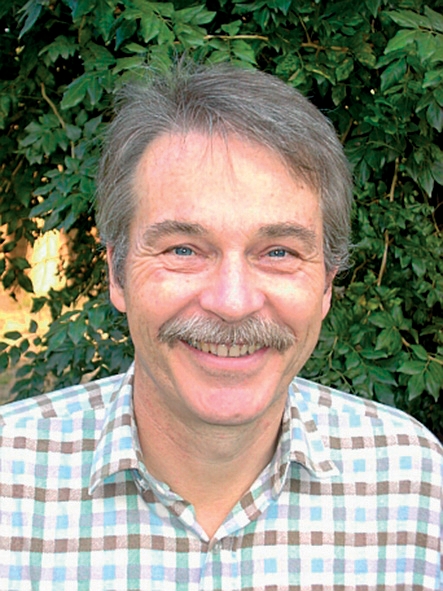


Small incision cataract surgery (SICS) is one of the cataract surgical techniques commonly used in developing countries. This technique usually results in a good visual outcome and is useful for high-volume cataract surgery.^1^^,^^2^^,^^3^

This article describes how to minimise surgical complications in SICS.

## Before you begin

With SICS, as with all cataract surgery techniques, it is mandatory to perform a thorough preoperative assessment of the patient (see article on page 12). This will allow the surgeon to prepare for anticipated complications – for example, a dislocated or subluxated lens – and to plan the operation accordingly.

Prepare the patient in the following way:

- Wash the patient's face.
- Instil povidone-iodine (Betadine) 5% aqueous eye drops (Figure 1).
  **Figure 1 - Before you begin:**
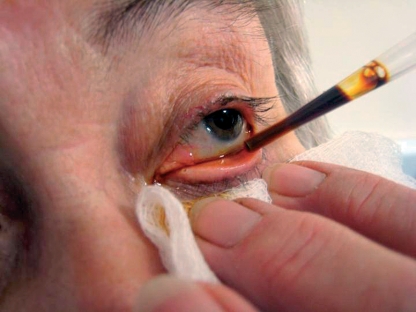
***Instilling povidone-iodine (Betadine) 5% eye drops***
- Clean the skin around the eye with povidone-iodine 10% (Figure 2).
  **Figure 2 - Before you begin:**
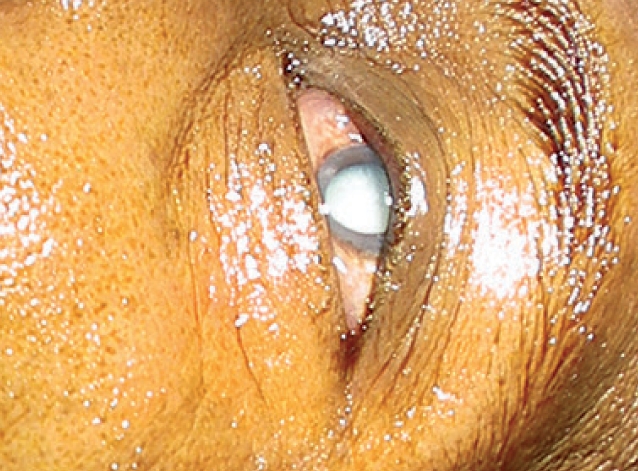
***Skin around the eye cleaned with povidone-iodine (Betadine) 10%***

Other measures will also help to reduce the risk of postoperative endophthalmitis: proper hand washing (see ‘how to’ article on page 17), the use of sterile instruments, the ‘non-touch’ technique, the subconjunctival injection of antibiotics,^4^^,^^5^ and the intracameral injection of cefuroxime^6^ at the end of surgery (see article on page 11). The dose of intracameral cefuroxime must be meticulously prepared, as no commercially made preparation is available (see box on page 11).

## Tunnel construction

### Tunnel size

Tunnel size The expected size and density of the nucleus should determine the size of the tunnel. For example, the extraction of immature cataracts in younger patients may only require a small tunnel, just large enough for the intraocular lens (IOL) optic to pass through. Very big, brown nuclei require a larger tunnel size. These nuclei can sometimes be up to 8 mm in diameter and 4 mm thick. However, a large tunnel need not be a problem: even larger tunnels are self-sealing and don't need suturing if they are prepared correctly. If there is doubt about the self-sealing effect, the surgeon may apply one or two sutures at the end of surgery. If correctly tied, these will, at the same time, reduce any induced astigmatism.

### Constructing the tunnel

- Only a correct sclerocorneal tunnel incision, at least 1 to 2 mm into the clear cornea, leads to a self-sealing wound.
- Scleral cauterisation before tunnel. construction reduces the risk of pre- and postoperative hyphaema.
- Sharp tunnel instruments (such as the crescent knife and keratome) should be used to construct the tunnel. A blunt keratome could cause stripping of Descemet's membrane.
- Stabilising the sclera with toothed forceps makes tunnel construction easier (Figure 3). However, in order to avoid tunnel damage and leakage, the forceps should not be used on the tunnel flap.
  **Figure 3 - Tunnel construction:**
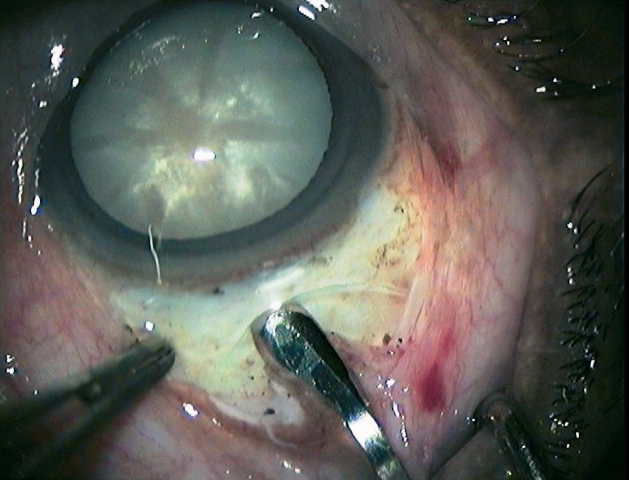
***Stabilising the sclera with toothed forceps***
- With a half-thickness sclerocorneal tunnel incision, the direction of the crescent knife should always be parallel to the sclerocorneal plane.
- Judge the depth of half-thickness sclerocorneal tunnel incisions by observing how clearly you can see the crescent knife during the incision (Figure 4). If the crescent knife can be seen very clearly, this indicates that the scleral layer is very thin and that the crescent knife might perforate to the outside. (causing what is known as a ‘buttonhole’)
  **Figure 4 - Tunnel construction:**
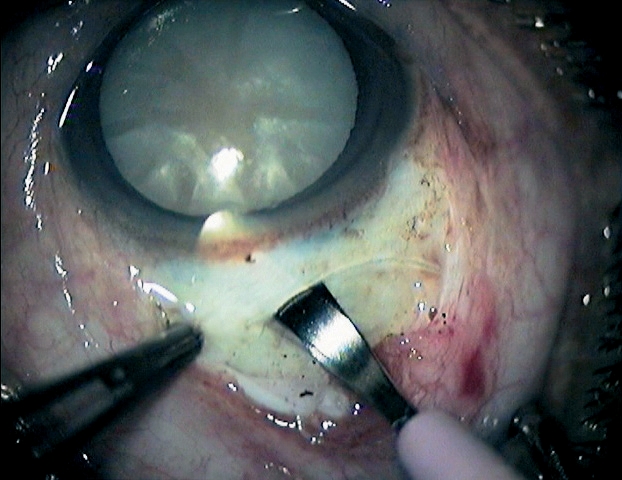
***Making the sclerocorneal tunnel incision***
- A buttonhole can be corrected by making a deeper ‘frown’ incision and dissecting the tunnel in a deeper plane, starting at the opposite side of the buttonhole.^7^
- If the crescent knife is not visible during the incision, this indicates that you are working too deeply inside the sclera; you may perforate towards the anterior chamber's angle (a ‘premature entry’).
- A premature entry could lead to surgical complications, such as iris trauma or iridodialysis, iris prolapse, and a tunnel which is not self-sealing.
- Manage a premature entry by starting a more shallow dissection at the other end of the tunnel. Suturing of the wound is required at the end of surgery.^7^

## Opening of the anterior capsule

**Figure 5 - Opening of the anterior capsule:**
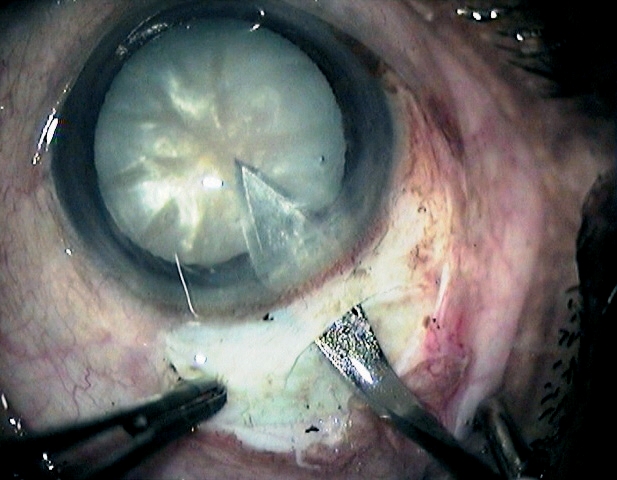
***Linear capsulotomy***

This can be done by different techniques (such as linear capsulotomy (Figure 5), the ‘can-opener’ technique, and triangular or V-shaped capsulotomy) or by capsulorhexis.

Capsulotomies are easy to perform, but may lead to uncontrolled capsular tear extension, posterior capsule rupture, vitreous loss, and IOL decentration. These problems can be avoided by a careful hydrodissection, especially in patients with posterior polar cataract or posterior lenticonus (hydrodissection is most effective if the fluid is injected directly into the capsule^7^). Keeping instrument manipulation to a minimum during surgery will also help you to avoid posterior capsule rupture.

**Figure 6 - Opening of the anterior capsule:**
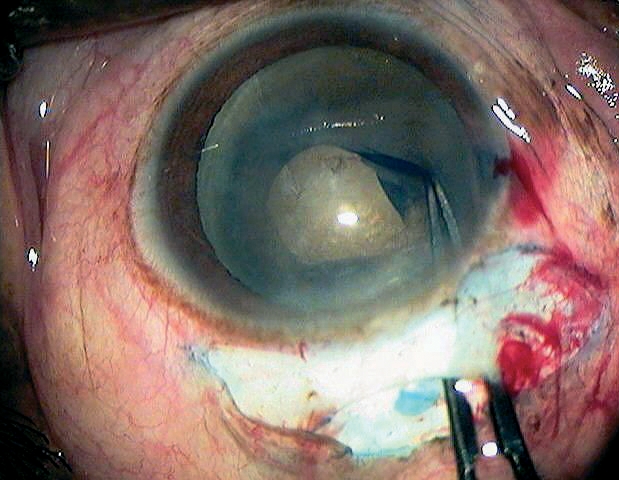
***Continuous curvilinear capsulorhexis***

The best capsular opening is a continuous curvilinear capsulorhexis (CCC): it will guarantee a long-term, ‘in the bag’ IOL centration (Figure 6). However, CCC is more difficult to learn. This technique sometimes requires staining of the capsule and the opening also needs to be large enough for the nucleus to get through. It may therefore not be possible to use this technique in eyes with very big nuclei and smaller pupils. In such a situation, a linear, triangular, or other capsulotomy may be preferred.

Failing to complete the anterior capsulotomy, making a too-small CCC, and pulling residual anterior capsular tags can cause the posterior capsule to rupture. Early recognition and correction of these problems is very important to avoid further complications.

## Nucleus removal

During SICS, different techniques can be used to remove the nucleus: either hydroexpression alone (using an anterior chamber maintainer), hydroexpression plus extraction (using an irrigating vectis or Simcoe cannula), or extraction alone (using a ‘fishhook’ needle). Problems with these different SICS techniques are mainly related to the size of the tunnel and the proximity of the nucleus to the corneal endothelium.

**Figure 7 - Nucleus removal:**
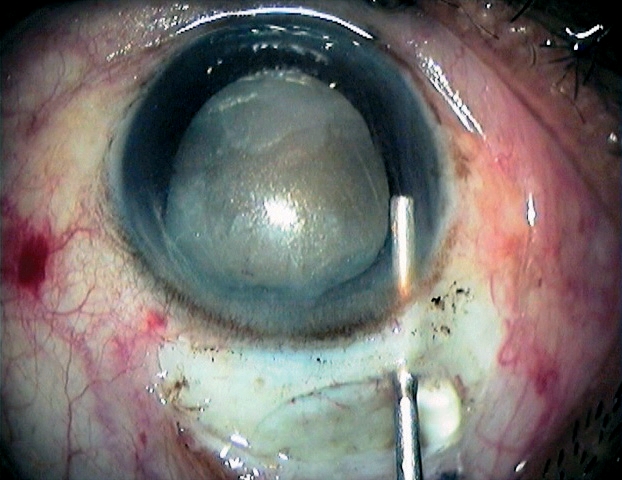
***Checking the tunnel size***

Difficulties with nucleus delivery are mostly due to the inner tunnel opening being too small. This should be checked before nucleus removal, e.g. with the visco cannula (Figure 7). If there is any doubt about the correct tunnel size, it is better to further enlarge the tunnel before removing the nucleus. However, the surgeon should avoid cutting into the anterior chamber's angle while enlarging the inner tunnel opening, as this carries an increased risk of hyphaema.

**Figure 8 - Nucleus removal:**
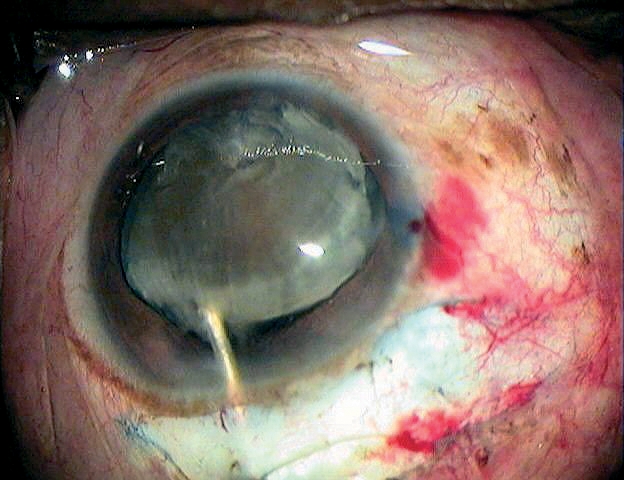
***Mobilising the nucleus before lifting it into the anterior chamber***

While lifting the nucleus into the anterior chamber (Figure 8), special care is required in patients with pseudoexfoliation and in older patients with weak zonules.

While delivering the nucleus through the tunnel, accidental contact between the nucleus and the corneal endothelium must be avoided. Otherwise, postoperative corneal oedema, and sometimes even corneal decompensation, may occur.

**Figure 9 - Nucleus removal:**
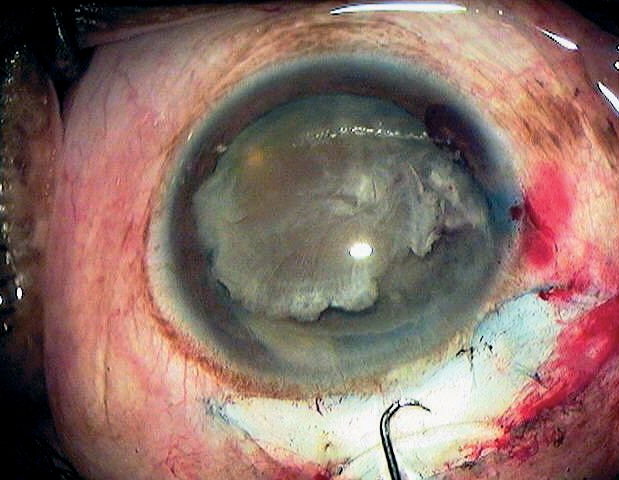
***Inserting the fishhook needle***

**Figure 10 - Nucleus removal:**
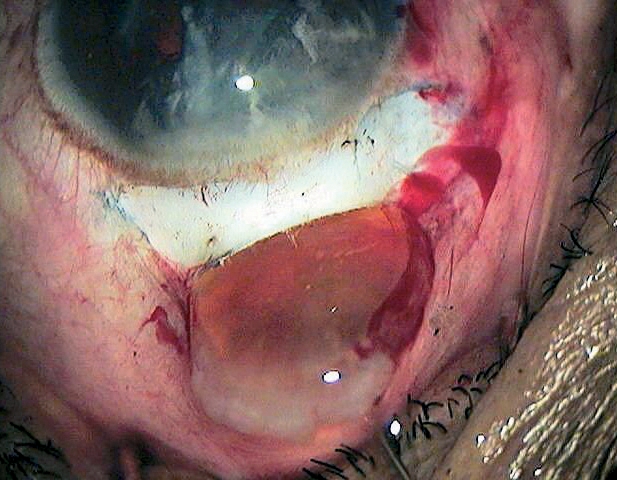
***Delivering the nucleus***

In order to avoid such corneal problems, you must inject sufficient viscoelastic fluid between the lens and the cornea to protect the endothelium. Instruments for nucleus removal, such as the irrigating vectis, Simcoe cannula, or fishhook, should be kept away from the cornea and should not push the nucleus against the cornea during nucleus delivery.These instruments should push slightly posteriorly, which will help to open the incision for easier nucleus delivery (Figures 9 & 10). In addition, gently pulling the bridle suture makes nucleus delivery through the tunnel easier.

## Removal of the cortex

**Figure 11 - removal of the cortex:**
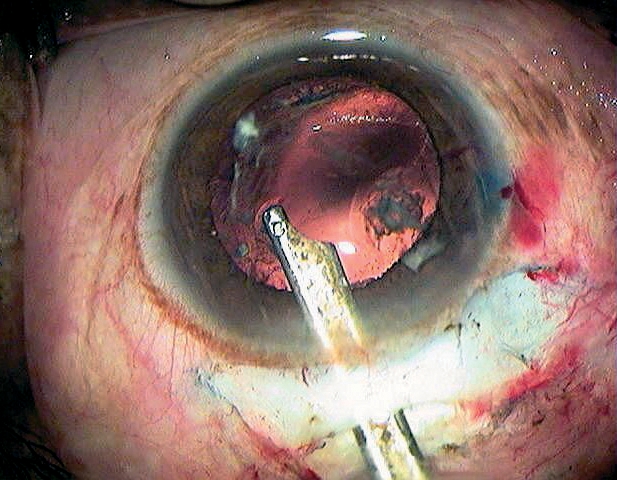
***Removing the cortex using a Simcoe cannula***

Most of the lens cortex can be removed with a Simcoe cannula through the tunnel (Figure 11). A sub-incisional cortex can be safely aspirated through a side port at 130–180˚ from the incision site.^8^ If stripping of Descemet's membrane occurs while cleaning the cortex, great care should be taken not to tear it off. If this happens, air should be injected into the chamber at the end of the operation to push Descemet's membrane against the cornea.

While clearing the cortex with a Simcoe cannula, posterior capsule rupture and vitreous loss may occur. This can be avoided by carefully watching the posterior capsule. Wrinkles indicate that the posterior capsule is caught in the aspiration port of the Simcoe cannula. This requires immediate backflushing to avoid posterior capsular rupture.

To reduce the risk of a postoperative increase in intraocular pressure, thorough removal of viscoelastics is required.
